# Supplementary material for: Isolated BAP1 Genomic Alteration in Malignant Pleural Mesothelioma Predicts Distinct Immunogenicity with Implications for Immunotherapeutic Response
Source: Cancers (Basel). 2022 Nov 16;14(22):5626. doi: 10.3390/cancers14225626 (PMC9688367; doi:10.3390/cancers14225626)
Supplement: Supplementary file 1 [file cancers-14-05626-s001.zip › cancers-1971981-supplementary.pdf]

Supplementary Materials

Figure S1: Comparison of immune checkpoint gene mRNA expression levels as a function of TSG genotypes in 86 MPM samples from the TCGA cohort.

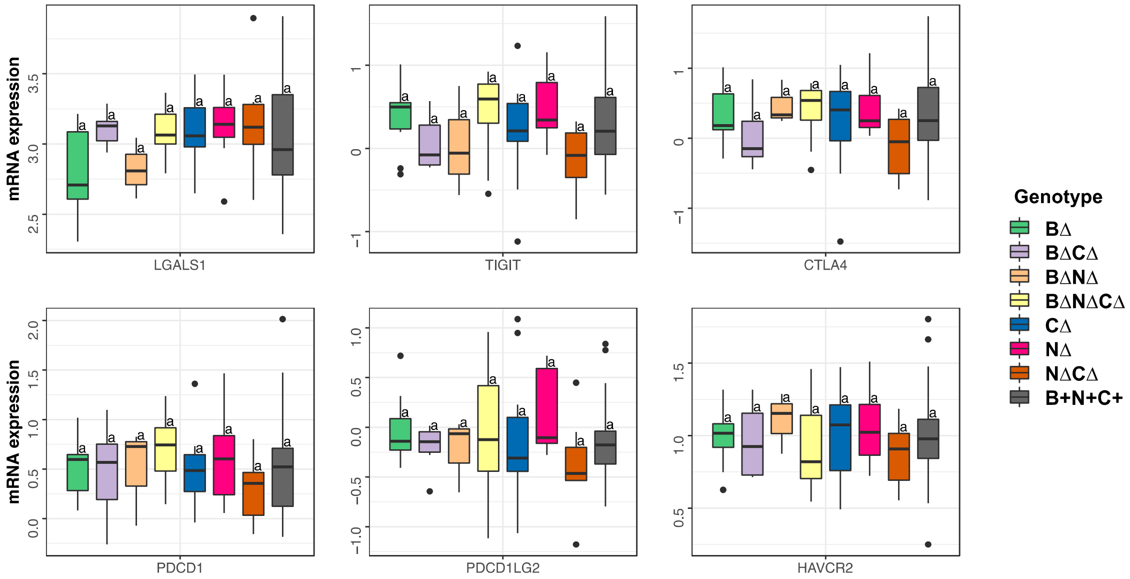

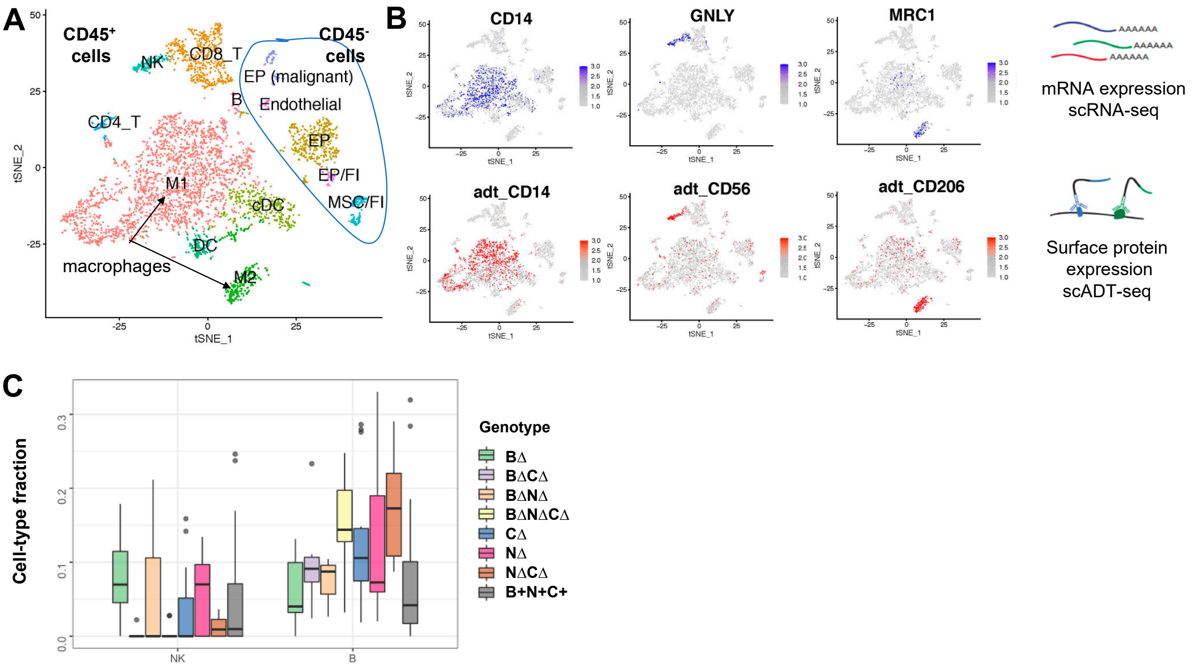

Figure S2: CIBERSORTx analysis of TCGA MPM RNA-seq dataset with MPM scRNA-seq reference of ten major cell types. **(A)** Transcriptome-based clustering of 4,912 CITE-seq single-cell expression profiles of malignant pleural mesothelioma (MPM)[1] reveals distinct cell populations. Cell types can be discerned by marker gene expression. B, B cells; CD4 T, $CD4^+$ T cells; CD8 T, $CD8^+$ T cells; NK, natural killer cells; M1, M1-like Macrophages; M2, M2-like Macrophages; DC, dendritic cells; cDC, conventional DC; EP (malignant), epithelial malignant cells; Endothelial; EP, epithelial; EP/FI, epithelial and fibroblast; MSC/FI, mesenchymal stem cell and fibroblast. **(B)** mRNA (blue) and corresponding antibody-derived tag (ADT) (red) signal for the CITE-seq antibody panel projected on the t-SNE plot from a subset of canonical markers representative of annotated cells. **(C)** Boxplots of the relative cell fractions of NK and B cell types for each sample estimated by CIBERSORTx.

Table S1: Comparison of pleural mesothelioma histological subtypes as a function of TSG genotypes in 86 MPM samples from the TCGA cohort.

Genotype	Epithelioid	Non-epithelioid		
		Biphasic	Diffuse malignant	Sarcomatoid
B+N+C+	19	6	0	1
BΔ	9	2	0	0
NΔ	6	0	1	0
CΔ	9	5	1	1
BΔNΔ	0	2	1	0
BΔCΔ	4	0	2	0
NΔCΔ	3	4	0	0
BΔNΔCΔ	7	4	0	0

Table S2: Comparison of pleural mesothelioma histological subtypes as a function of TSG genotypes in 61 MPM samples from the MSK-IMPACT cohort.

Genotype	Epithelioid	Biphasic	Sarcomatoid	Not specified
B+N+C+	9	0	1	10
BΔ	10	0	1	9
NΔ	1	0	0	1
CΔ	2	1	0	2
BΔNΔ	3	1	0	2
BΔCΔ	2	0	0	1
NΔCΔ	0	1	0	2
BΔNΔCΔ	0	1	0	1

Table S3: Multivariate cox regression analysis based on *BAP1*, *NF2* and *CDKN2A/B* status for TCGA MPM dataset

Alteration	HR	95% CI	P-value
BAP1	0.6901	0.4127 - 1.154	0.157
NF2	1.4767	0.8718 - 2.501	0.147
CDKN2A/B	3.2285	1.892 - 5.509	1.72E-05

Table S4: Multivariate cox regression analysis based on *BAP1*, *NF2* and *CDKN2A/B* status for MSK-IMPACT dataset

Alteration	HR	95% CI	P-value
BAP1	0.6489	0.2864 - 1.471	0.30019
NF2	0.5104	0.1485 - 1.754	0.28562
CDKN2A/B	5.3113	1.7022- 16.572	0.00402

Table S5: Genes for Pemetrexed response signature [2]

TYMS	*NDC80*	*DEPDC1*	*TTK*	*PRIM1*	*RFC4*
UBE2C	*CENPF*	*KIF20A*	*CENPA*	*DTL*	
TOP2A	*TPX2*	*ASPM*	*KIF15*	*NUSAP1*	
NUF2	*RAD51AP1*	*EZH2*	*MCM2*	*KIF11*	

Table S6: Genes for Palbociclib response signature [3]

ANLN	*CDCA8*	*FAM83D*	*MND1*	*MYBL2*	*RAD54L*
ARHGAP11A	*CDKN3*	*FANCI*	*MTFR2*	*NCAPH*	*RRM2*
ASF1B	*CDT1*	*FOXM1*	*MYBL2*	*NDC80*	*SGOL1*
ASPM	*CENPA*	*GTSE1*	*KIF14*	*NEIL3*	*SKA1*
AUNIP	*CENPE*	*KIF11*	*KIF15*	*NUF2*	*SKA3*
AURKA	*CENPI*	*KIF14*	*KIF20A*	*NUSAP1*	*SPC25*
AURKB	*CENPM*	*KIF15*	*KIF23*	*OIP5*	*STIL*
BIRC5	*CENPN*	*KIF20A*	*KIF2C*	*ORC1*	*TICRR*
BLM	*CENPO*	*KIF23*	*KIF4A*	*ORC6*	*TPX2*
BUB1	*CENPW*	*KIF2C*	*KIF4B*	*PIF1*	*TRIP13*
BUB1B	*CEP55*	*KIF4A*	*KIFC1*	*PKMYT1*	*TROAP*
CCNA2	*CHEK1*	*KIF4B*	*MCM10*	*PLK1*	*TTK*
CCNB2	*CLSPN*	*KIFC1*	*MCM4*	*POLQ*	*UBE2C*
CDC20	*DEPDC1*	*MCM10*	*MCM7*	*PRC1*	
CDC25A	*DEPDC1B*	*MCM4*	*MELK*	*PTTG1*	
CDC45	*DLGAP5*	*MCM7*	*MKI67*	*PTTG2*	
CDCA3	*EXO1*	*MELK*	*MND1*	*PTTG3P*	
CDCA5	*FAM64A*	*MKI67*	*MTFR2*	*RAD51*	

Table S7: Genes for anti-PD-1 resistance signature [4]

#	Gene symbol	P-value	Fold change (log2)	#	Gene symbol	P-value	Fold change (log2)
1	A1CF	0.001926017	-1.760698572	80	MGC16121	0.00756817	-1.613353038
2	ABL2	0.005778448	1.333253888	81	MUC5B	0.008544592	1.142758193
3	ACADM	0.002468075	-1.726467137	82	MUM1L1	0.006036535	1.526545127
4	ACBD5	0.007178588	1.248070266	83	MUS81	0.00388895	-1.742211687
5	ADAM6	0.001893221	-1.834722179	84	MX1	0.004426645	-1.453808943
6	ADAMTS19	0.004475869	1.686879384	85	MYO7A	0.000303608	1.851147905
7	ADRA1A	0.005377473	1.580804029	86	NBPF10	0.00885333	1.627612367
8	AIM2	0.002310882	-1.858689042	87	NEUROG3	0.00607667	-1.769533968
9	ALDH1A2	0.007933211	-1.688858537	88	NFATC2IP	0.002312654	-1.68286809
10	ARGFXP2	0.004345199	-1.729656818	89	NFATC3	0.001590321	1.784144289
11	BBS7	0.006448138	1.790803639	90	NOL9	0.005270846	1.688052195
12	BCL7A	0.003063777	1.874429641	91	NOTCH2NL	0.006364424	1.673785308
13	BEND4	0.004932073	-1.661526942	92	NUP54	0.001403774	1.738998508
14	BRCA1	0.009562223	-1.145010704	93	OPRD1	0.004332597	-0.781215631
15	C16orf42	0.000197024	-1.582592059	94	OR10A7	0.003273514	-1.87472641
16	C1orf127	0.00355508	-1.254142722	95	OR2A2	0.003984551	1.76912989
17	CDC6	0.009232455	1.617865007	96	OR6C65	0.002509217	-1.569785762
18	CEACAM20	0.006254206	1.686429388	97	PANK2	0.003087108	-1.796296116
19	CLDN6	0.006538255	-1.291318325	98	PCBP2	0.003637452	1.702620618
20	CNRIP1	0.008961682	1.778101031	99	PCGF2	0.009542674	1.452956756
21	COG3	0.006451243	1.803291288	100	PCNA	0.002972182	1.846747128
22	COL9A1	0.005722518	-1.617914118	101	PGM3	0.006890908	1.664639855
23	CRISP2	0.003835624	-1.447575061	102	PHF12	0.008226558	1.496812962
24	CST4	0.001589578	1.366581633	103	PNPO	0.002177928	-1.896611723
25	CYFIP1	0.001372974	1.850185935	104	PPP2R2B	2.31528E-05	2.015234651
26	DBN1	0.000386029	1.704212311	105	PTK2B	0.006597816	-1.729999651
27	DEFA5	0.008386785	-1.211668569	106	RAB43	0.003885702	-1.809824223
28	DNAH17	0.005607152	-1.464095745	107	RARRES1	0.003935857	-1.614961121
29	DNAJA3	0.008609746	-1.7616982	108	RBMY3AP	0.002218685	1.731480297
30	EBAG9	0.008616486	-1.687761246	109	RIMBP3C	0.007058752	1.786474302
31	EIF5	0.003967313	-1.536055051	110	RNF39	0.006316426	0.56780024
32	ENAH	0.009775625	1.626404245	111	RPS6KA5	0.00876094	-1.473703717
33	EPB41	0.005642849	1.358590261	112	RTN3	4.07388E-05	-1.684739112
34	ERAS	0.00047968	-1.861129863	113	SCNN1A	0.006542277	-1.245391428
35	EYA4	0.002490164	-1.532018618	114	SCP2	0.001485885	-1.600593211
36	FAM71E2	0.001453467	-1.768660384	115	SERPINB12	0.004367128	1.826590025
37	FBXO24	0.001154684	-1.727137536	116	SIRT5	0.007170287	-1.523277771
38	FLJ14107	0.0074351	1.575618262	117	SKA2	0.003611884	-1.659612421
39	FLJ25328	0.002024152	1.347527517	118	SLC37A3	0.008096465	1.639160339
40	GAB2	0.008077743	-1.66290649	119	SLC4A8	0.002591918	-1.457279481
41	GABRG2	0.003301371	-1.596806546	120	SLC6A14	0.004968129	-1.547661562

42	GAS1	0.002334935	1.815942178	121	SLCO1A2	0.008498208	1.639442258
43	GCET2	0.001721565	-1.920599081	122	SNCB	0.004915308	1.760880392
44	GLE1	0.009775182	1.47130706	123	SNORA5A	0.009219678	-1.741713837
45	GPM6B	0.005833483	1.794201554	124	SNORD49A	0.002132703	1.396865827
46	GPR55	0.005585241	-1.349744764	125	SNORD69	0.002274865	1.81096929
47	H6PD	0.008329606	1.557431585	126	SNX20	0.001129844	1.083066757
48	HIST1H2BM	0.005062682	1.68876799	127	SP110	0.003811201	-1.624202989
49	HIVEP3	0.005513168	-1.470160141	128	SP140	0.004311164	-1.482442321
50	HPS4	0.009293869	-1.655441066	129	SPATA7	0.008893923	1.747944822
51	HSPA1L	0.005354921	-1.800870381	130	SPOCD1	0.007706922	-1.523008143
52	IGLL1	0.002762734	-1.727313285	131	SPTBN4	0.009027349	-1.201780935
53	IL22RA2	0.005159646	1.766686677	132	SRMS	0.006774626	-1.446907584
54	IL28A	0.003103524	-1.810579399	133	ST3GAL6	0.00154254	-1.474764453
55	IL33	0.002354446	1.493344196	134	STOML1	0.006918576	-1.589023659
56	INPP4B	0.000701043	-1.454378385	135	STXBP1	0.003932145	1.575588331
57	IP6K2	0.007251889	1.440431504	136	TAAR6	0.007863254	-0.661371267
58	IQCA1	0.000364482	1.803086712	137	TAC4	0.006791879	-1.512292307
59	IRF7	0.005788122	-1.649308796	138	TBPL2	0.005093235	-1.34782936
60	ITGA11	0.009793455	1.550256145	139	TCP11L2	0.000416908	1.969953914
61	KCNF1	0.00733989	1.508769074	140	TEX9	0.006979143	1.804585709
62	KCNMB2	0.003413733	1.348292525	141	TGFB2	0.003352966	1.69758668
63	KCNQ2	0.002604519	-1.899825014	142	TGM5	0.006334409	1.812920903
64	KIAA0319L	0.007254819	-1.713436396	143	TIAM2	0.009361282	-1.512486564
65	KIF16B	7.50913E-05	2.036376982	144	TINAGL1	0.00273972	1.824394479
66	KLF11	0.004597693	1.583933769	145	TMEM140	0.003795543	-1.596553115
67	KLHL6	0.004536732	1.74601748	146	TMEM170A	0.006249256	-1.655719188
68	KLRG2	0.00971813	-1.347708588	147	TRIM17	0.00870819	1.623783169
69	LDHA	0.006152084	-1.72956763	148	TRPV1	0.000210797	-1.985760569
70	LRRC1	0.004125048	-1.827036608	149	UBE3C	0.008646868	-1.718827377
71	LRRC34	0.002050979	1.497367526	150	UBQLN1	0.006427152	1.137157331
72	M6PR	0.005673107	-1.623539694	151	USP17	0.005235872	-1.27888599
73	MAN1A2	0.003284545	-1.844261328	152	VCL	1.68493E-05	1.919701923
74	MAPK1IP1L	0.002582006	-1.807536403	153	WIPF2	0.002724628	-1.789207042
75	MAX	0.0099241	-1.707248148	154	WNT1	0.005650741	1.574307092
76	MBP	0.002586935	-1.803133409	155	WNT5B	0.00546917	-0.935186586
77	MCRS1	0.002048342	1.162421057	156	WWOX	0.000635214	1.683374
78	METTL7B	0.006035269	-1.667458541	157	ZNF45	0.007844801	1.450532206
79	MEX3B	0.008433225	1.631341381	158	ZNF74	0.003772907	-1.644150598

Table S8: The anti-PD-1-resistant mRNA signature was used to predict the subgroups. TCGA MPM tumors predicted to be anti-PD-1-sensitive tumors were enriched in samples with *BAP1* loss only. A two-sided proportion test was used to compare each genotype to the B+N+C+ group.

Genotype	Responsive	Resistant	P-value
B+N+C+	9<br>(epithelioid)	10<br>(3 biphasic, 6 epithelioid, 1 sarcomatoid)	1
BΔ	11<br>(2 biphasic, 9 epithelioid)	0	0.0032
NΔ	4<br>(Diffuse malignant, 3 epithelioid)	3<br>(epithelioid)	0.6584
CΔ	3<br>(2 biphasic, 1 epithelioid)	9<br>(3 biphasic, 5 epithelioid, 1 sarcomatoid)	0.2130
BΔNΔ	0	2<br>(biphasic)	0.1978
BΔCΔ	3<br>(1 diffuse malignant, 2 epithelioid)	3<br>(1 diffuse malignant, 2 epithelioid)	0.9104
NΔCΔ	0	7<br>(4 biphasic, 3 epithelioid)	0.0243
BΔNΔCΔ	3<br>(1 biphasic, 2 epithelioid)	6<br>(2 biphasic, 4 epithelioid)	-

Table S9: Candidate TF regulators (5% FDR) based on **Fig 4A**. Functional annotations were determined from terms overrepresented from the canonical pathway and from the Gene Ontology 'Biological Process' gene sets associated with the candidate regulator based on ISMARA analysis.

Candidate regulator	Ontologies associated with geneset
**IRF2_STAT2_IRF8_IRF1**	Type I interferon (alpha/beta IFN) Pathway
**IRF9**	CXCR3-mediated signaling events, type I interferon (alpha/beta IFN) pathway
**IRF3**	Type I interferon (alpha/beta IFN) Pathway
**BCL6B**	Syndecan-4-mediated signaling events
**AR_NR3C2**	AP-1 transcription factor network

References

1. Ma X, Somasundaram A, Qi Z, Hartman DJ, Singh H, Osmanbeyoglu HU. SPaRTAN, a computational framework for linking cell-surface receptors to transcriptional regulators. Nucleic Acids Res. 2021;49(17):9633-47. Epub 2021/09/10. doi: 10.1093/nar/gkab745. PubMed PMID: 34500467; PMCID: PMC8464045.

2. Hou J, Lambers M, den Hamer B, den Bakker MA, Hoogsteden HC, Grosveld F, Hegmans J, Aerts J, Philipsen S. Expression profiling-based subtyping identifies novel non-small cell lung cancer subgroups and implicates putative resistance to pemetrexed therapy. J Thorac Oncol. 2012;7(1):105-14. Epub 2011/12/03. doi: 10.1097/JTO.0b013e3182352a45. PubMed PMID: 22134068.
3. Malorni L, Piazza S, Ciani Y, Guarducci C, Bonechi M, Biagioni C, Hart CD, Verardo R, Di Leo A, Migliaccio I. A gene expression signature of retinoblastoma loss-of-function is a predictive biomarker of resistance to palbociclib in breast cancer cell lines and is prognostic in patients with ER positive early breast cancer. Oncotarget. 2016;7(42):68012-22. Epub 2016/10/18. doi: 10.18632/oncotarget.12010. PubMed PMID: 27634906; PMCID: PMC5356535.
4. Jang HJ, Truong CY, Lo EM, Holmes HM, Ramos D, Ramineni M, Lee JS, Wang DY, Pietropaolo M, Ripley RT, Burt BM, Lee HS. Inhibition of CDK4/6 Overcomes Primary Resistance to PD-1 Blockade in Malignant Mesothelioma. Ann Thorac Surg. 2021. Epub 2021/10/01. doi: 10.1016/j.athoracsur.2021.08.054. PubMed PMID: 34592265.
